# Supplementary material for: A unified model of human hemoglobin switching through single-cell genome editing
Source: Nat Commun. 2021 Aug 17;12:4991. doi: 10.1038/s41467-021-25298-9 (PMC8371164; doi:10.1038/s41467-021-25298-9)
Supplement: Supplementary file 1 — Supplementary Information [file 41467_2021_25298_MOESM1_ESM.pdf]

<u>Supplementary Information for:</u>

A unified model of human hemoglobin switching through single-cell genome editing

Yong Shen, Jeffrey M. Verboon, Yuannyu Zhang, Nan Liu, Yoon Jung Kim, Samantha Marglous, Satish K. Nandakumar, Richard A. Voit, Claudia Fiorini, Ayesha Ejaz, Anindita Basak, Stuart H. Orkin, Jian Xu, Vijay G. Sankaran*

*email: sankaran@broadinstitute.org

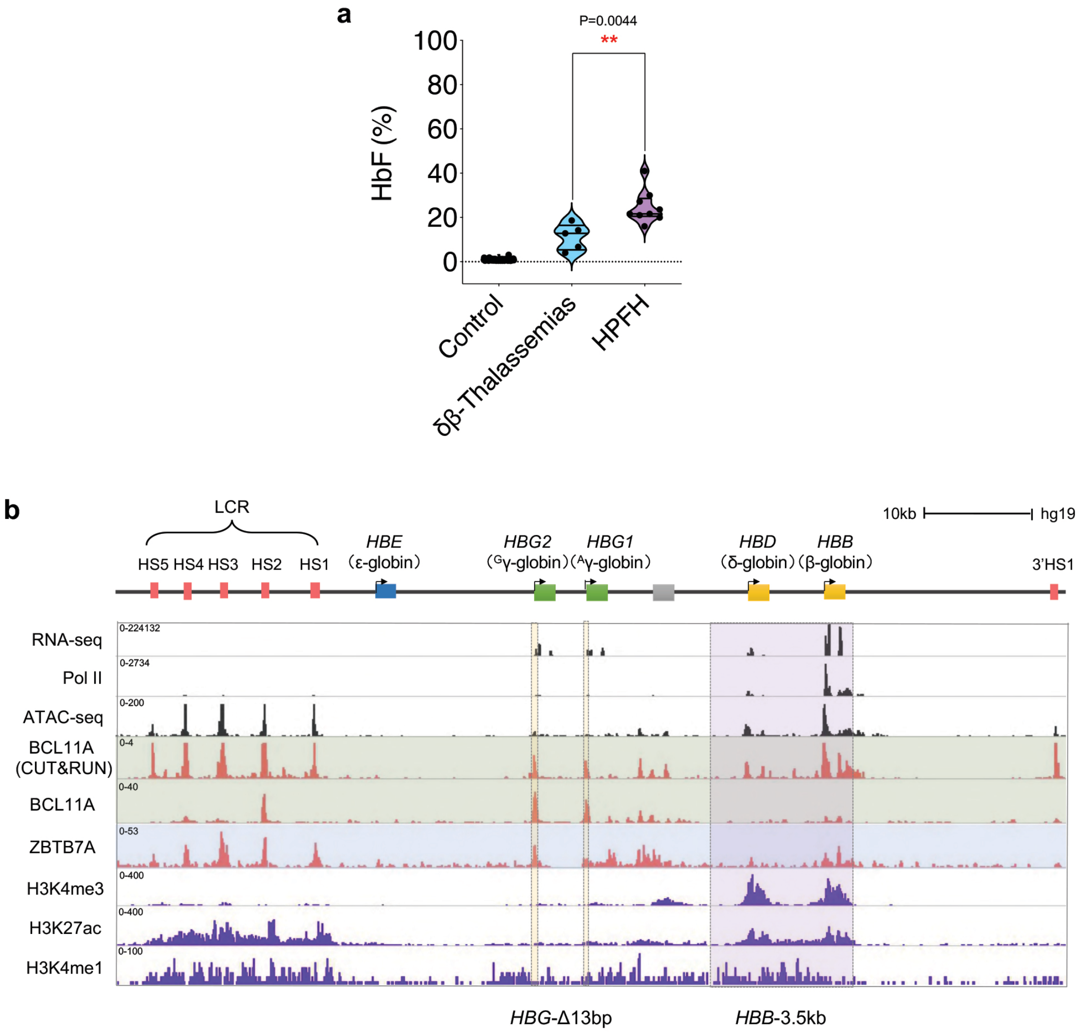

Supplementary Figure 1. Analysis of rare human genetic variation within the β-globin locus impacting HbF levels. a, Fetal hemoglobin levels in heterozygous individuals harboring large deletions within the β-globin locus that are either categorized as δβ-thalassemia (blue, n=5) or HPFH (purple, n=9) deletions depending upon the level of HbF production and other clinical parameters[11]. Results are shown as violin plots (P-values are labeled, **P<0.01 by a two-tailed Student t-test). **b,** Data showing the levels of RNA (RNA-seq), Pol II, chromatin accessibility (ATAC-seq), transcription factors BCL11A, ZBTB7A and histone modifications H3K4me1, H3K4me3, H3K27ac within the β-globin gene locus in human erythroblast cells. *HBB*-3.5kb: region starting from *HBD* upstream 3.5kb to *HBB* 3' end. Sequence Read Archive (SRA) accession numbers for RNA-seq, ATAC-seq, ChIP-seq, and CUT&RUN data are shown in Supplementary Data 2.

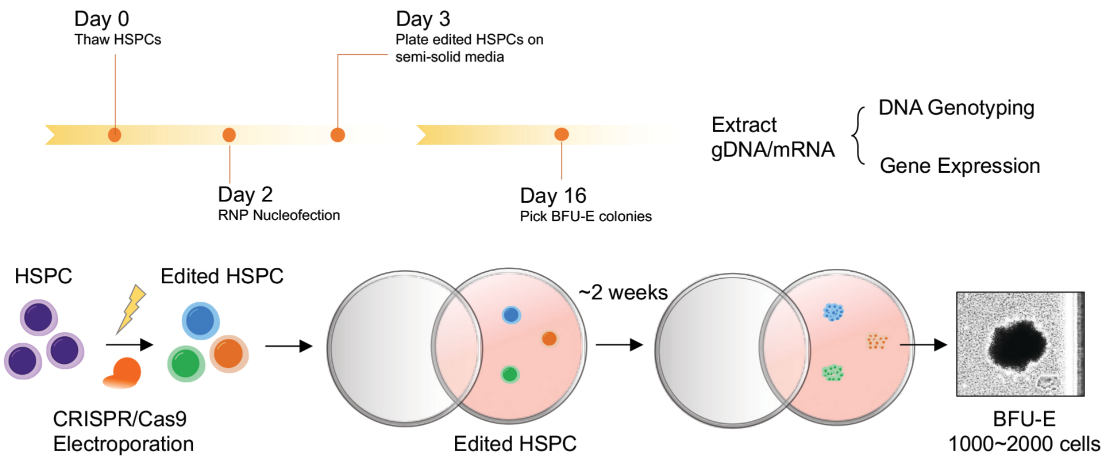

Supplementary Figure 2. Workflow of genetically targeted single-cell genome editing showing CRISPR/Cas9 delivery, colony forming cell assay and functional analysis. HSPC: human hematopoietic stem and progenitor cell; BFU-E: burst forming unit erythroid colony; gDNA: genomic DNA.

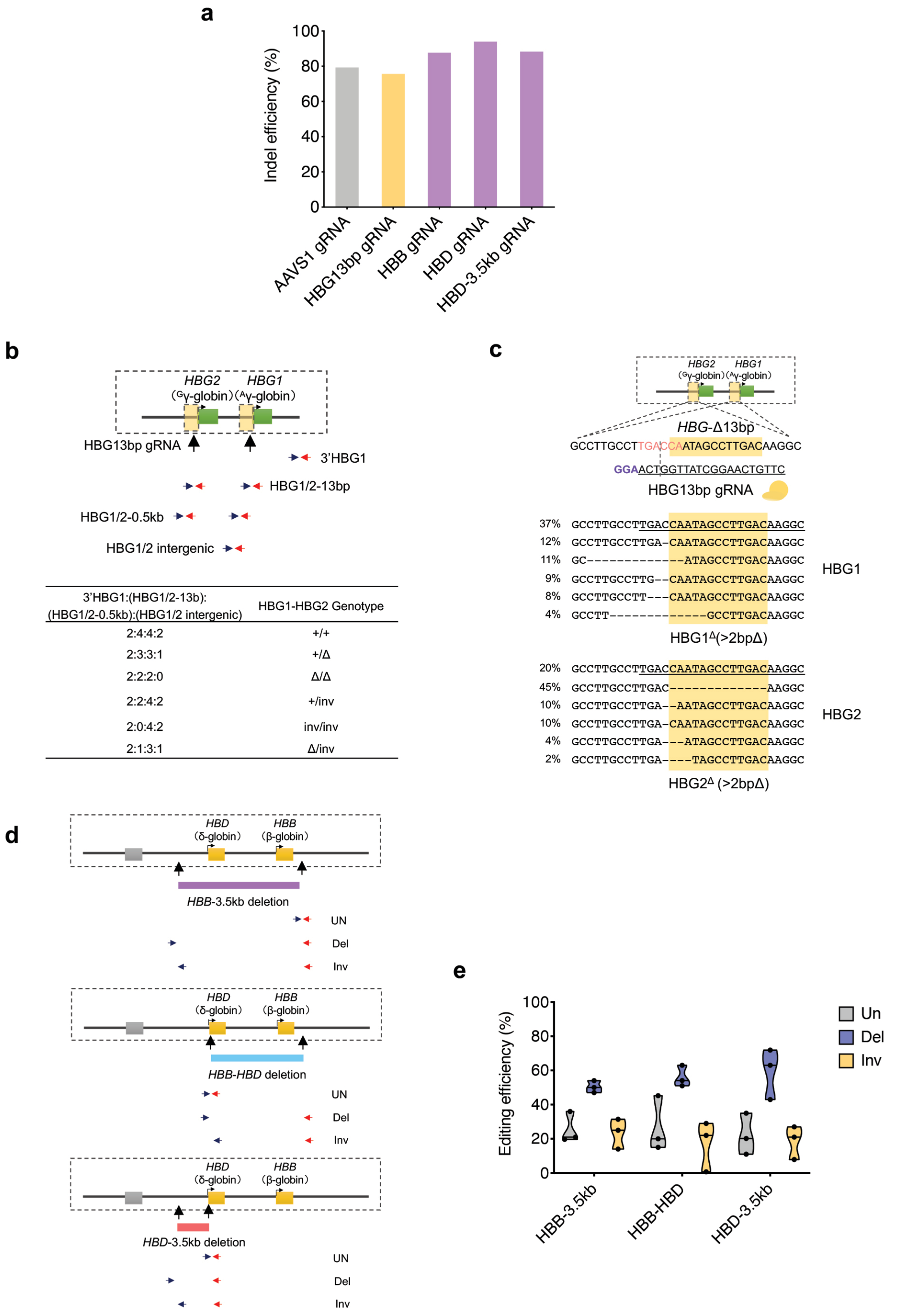

Supplementary Figure 3. Detection of genome editing events of specific cis-regulatory element perturbations. a, Editing efficiency of gRNAs evaluated by indel analysis in bulk HSPCs. **b,** Genotyping strategy of *HBG1-HBG2* genetic loci for genetic perturbations of *HBG*-Δ13bp based on qPCR amplification ratios of designated loci. **c,** Spectrum of mutations in bulk HSPCs upon genome editing of *HBG*-Δ13bp. **d,** Genotyping strategy of targeted genetic loci for genetic perturbations of *HBB*-3.5kb, *HBB-HBD*, and *HBD*-3.5kb regions. **e,** Deletion and inversion efficiency in bulk HSPCs for genetic perturbations of elements composing the entire *HBB*-3.5kb deletion, *HBB-HBD* deletion, and *HBD*-3.5kb deletion, n=3 biologically independent experiments. Results are shown as violin plots.

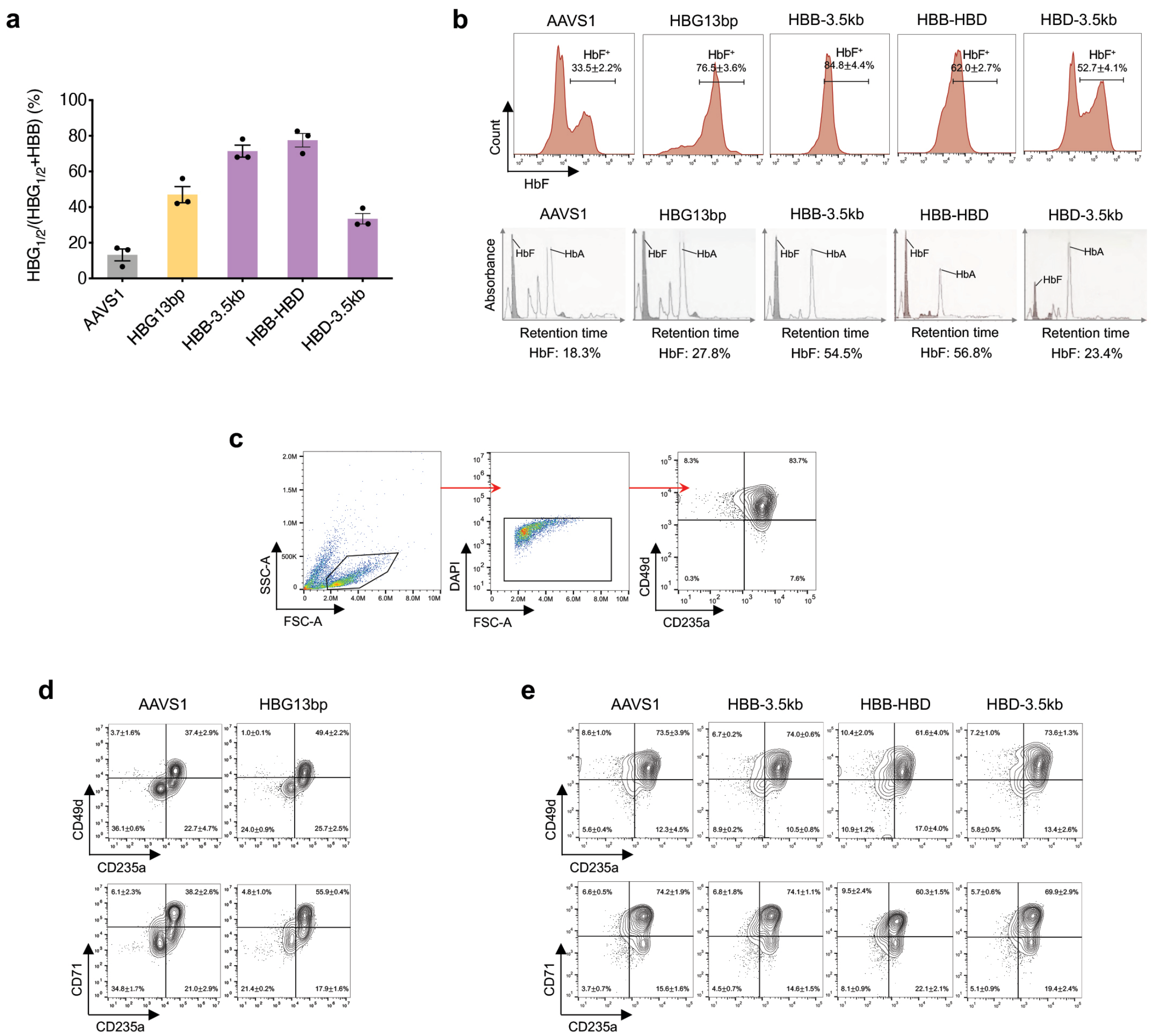

Supplementary Figure 4. Bulk population analysis through recapitulation of specific cis-regulatory element perturbations. a, RT-qPCR analysis of γ-globin mRNA for HSPC erythroblasts on day 12 of erythroid differentiation upon genetic perturbations of AAVS1, *HBG*-Δ13bp and *HBB*-3.5kb, *HBB-HBD* and *HBD*-3.5kb. **b,** HbF flow cytometry on day 13 of HSPC erythroid differentiation and HPLC on day 17. HbF: hemoglobin F (fetal form); HbA: hemoglobin A (adult form). HbF%: HbF as a fraction of HbA plus HbF. Results are shown as mean ± SEM from three biological replicates. **c,** A gating strategy employed for flow cytometric analyses. **d,** FACS analysis of CD49d, CD71 and CD235a erythroid markers for genetic perturbation of *HBG*-Δ13bp on day 13 of HSPC erythroid differentiation. Results are shown as mean ± SEM from three biological replicates. **e,** FACS analysis of CD49d, CD71 and CD235a erythroid markers for genetic perturbation of *HBB*-3.5kb, *HBB-HBD* and *HBD*-3.5kb on day 11 of HSPC erythroid differentiation. Results are shown as mean ± SEM from three biological replicates.

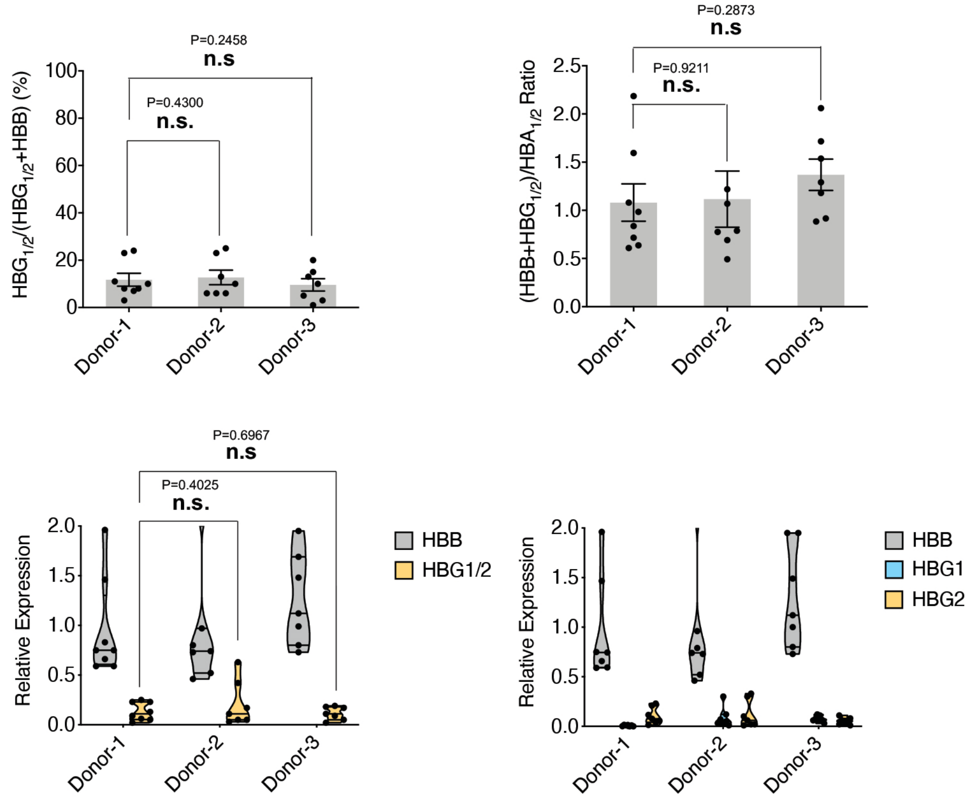

Supplementary Figure 5. Single cell-derived colony analysis of AAVS1 control. Globin gene expression analysis in BFU-E upon genetic perturbations of AAVS1 control gRNA in HSPCs from 3 different healthy donors, n=3 biologically independent experiments. Results are shown as mean ± SEM (P-values are labeled on the top of each comparison. n.s.: statistically non-significant by a two-tailed Student t-test).

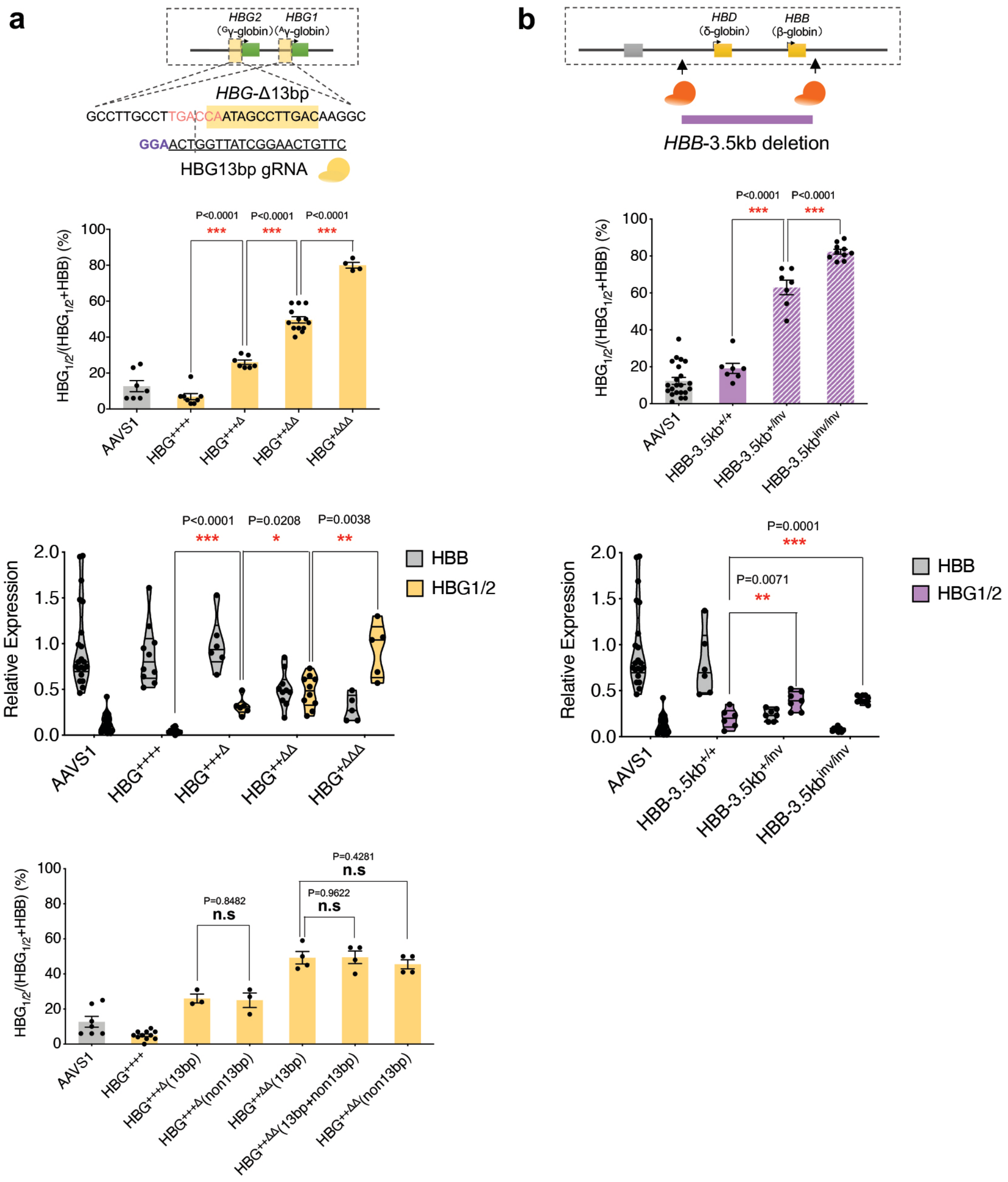

Supplementary Figure 6. Single cell-derived colony analysis through recapitulation of specific cis-regulatory element perturbations. a, Aggregate gene expression analysis for γ-globin (*HBG1/2*) and β-globin (*HBB*) mRNA in erythroid burst-forming units (BFU-E) derived from HSPC-derived erythroblasts upon genome editing of *HBG*-Δ13bp region in both promoters of *HBG1* and *HBG2,* n=3 biologically independent experiments. Results are shown as violin plot (P-values are labeled on the top of each comparison. *P<0.05, **P<0.01, ***P<0.001 by a two-tailed Student t-test). **b,** Globin gene expression analysis in BFU-E upon genetic perturbations of elements composing the *HBB*-3.5kb inversion. *HBB*-3.5kb^{inv}: region inversion starting from *HBD* upstream 3.5kb to *HBB* 3' end, n=6 biologically independent experiments. Results are shown as mean ± SEM (P-values are labeled on the top of each comparison. **P<0.01, ***P<0.001 by a two-tailed Student t-test).

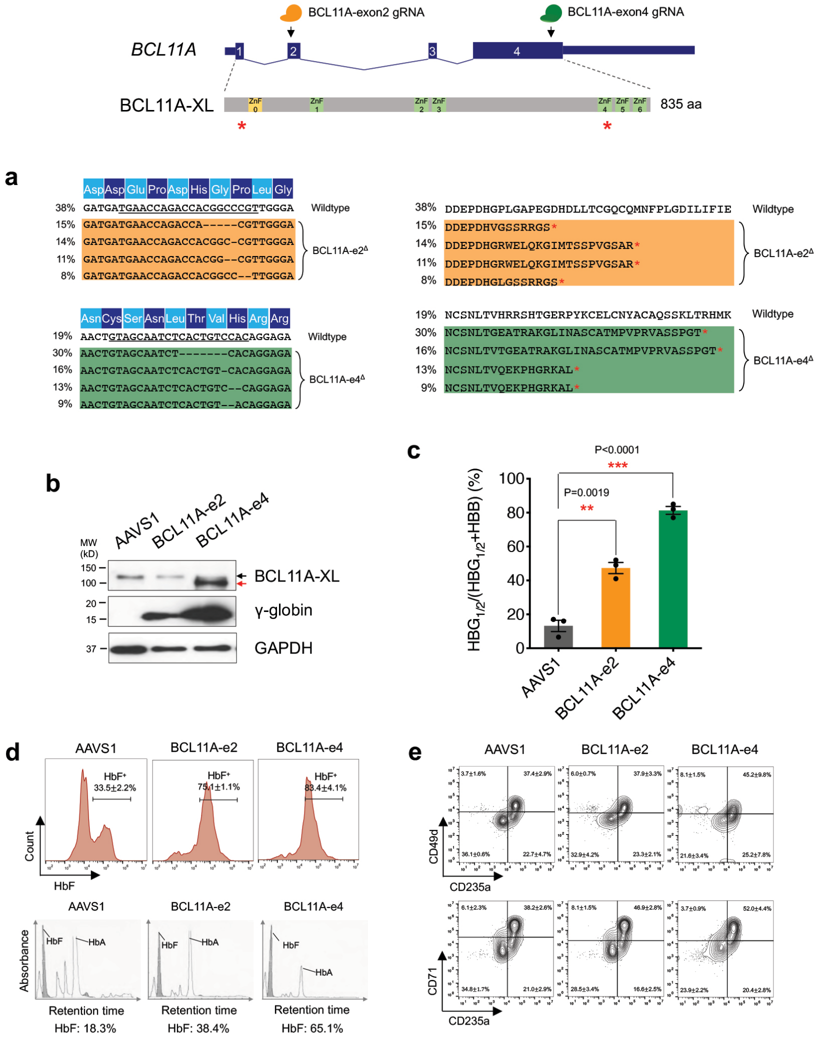

Supplementary Figure 7. Perturbation of BCL11A. a, Spectrum of genetic mutations and protein truncations in bulk HSPCs upon genome editing of BCL11A exon 2 and BCL11A exon 4. BCL11A-e2$^{\Delta}$: frameshift mutation in BCL11A exon 2; BCL11A-e4$^{\Delta}$: frameshift mutation in BCL11A exon 4. A red star indicates premature termination of protein elongation. **b,** Western blots showing the specificity of BCL11A, γ-globin, GAPDH antibodies in bulk HSPCs upon genome editing of AAVS1, BCL11A exon 2, and BCL11A exon 4. Black and red arrows indicate different protein molecular weights in gel electrophoresis. n=3 experiments were repeated independently with similar results. **c,** RT-qPCR analysis of γ-globin mRNA for HSPC erythroblasts on day 12 of erythroid differentiation upon genetic perturbations of AAVS1, BCL11A exon 2 and BCL11A exon 4. Results are shown as mean ± SEM from three biological replicates (P-values are labeled on the top of each comparison. **P<0.01, ***P<0.001 by a two-tailed Student t-test). **d,** HbF flow cytometry on day 13 of HSPC erythroid differentiation and HPLC on day 17. HbF: hemoglobin F (fetal form); HbA: hemoglobin A (adult form). HbF%: HbF as a fraction of HbA plus HbF. Results are shown as mean ± SEM from three biological replicates. **e,** FACS analyses of CD49d, CD71 and CD235a erythroid markers on day 13 of HSPC erythroid differentiation. Results are shown as mean ± SEM from three biological replicates.

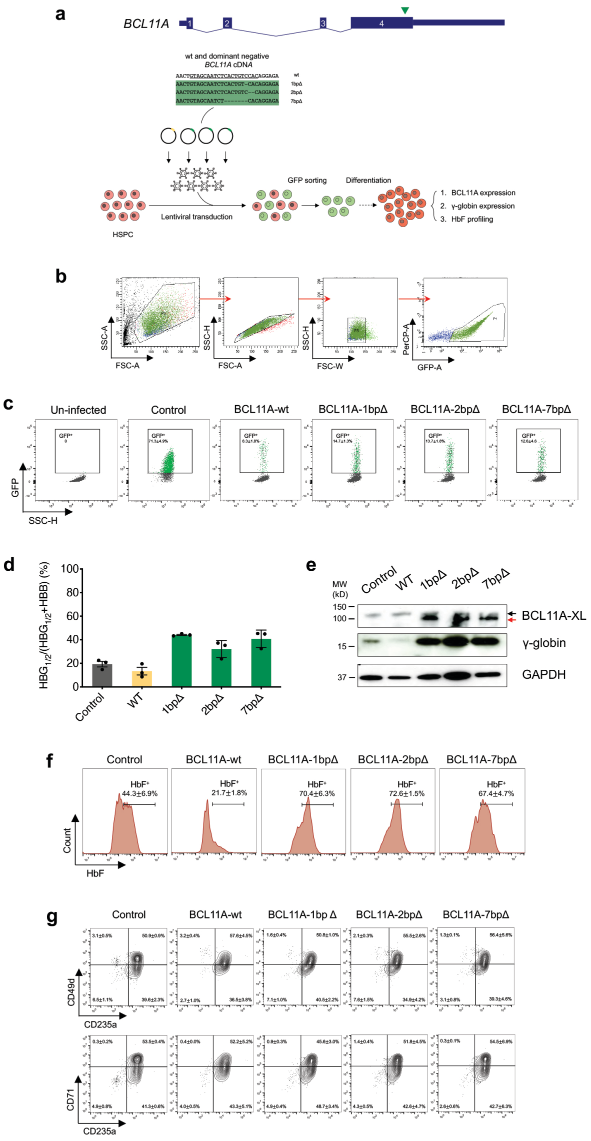

Supplementary Figure 8. Functional analysis of BCL11A exon 4 mutations. a, Workflow of exogenous expression of either the wild type or dominant negative mutant forms (1bpΔ, 2bpΔ and 7bpΔ) of the BCL11A XL in HSPCs showing lentiviral transduction, GFP sorting, erythroid differentiation and functional analysis. **b**, A sorting strategy for GFP positive cells from lentivirus infected population. **c,** GFP sorting of HSPC cells transduced with a co-transcribed GFP marker following an internal ribosome entry site after the *BCL11A* cDNA. Results are shown as mean ± SEM from three biological replicates. d**,** RT-qPCR analysis of γ-globin mRNA for HSPC erythroblasts on day 12 of erythroid differentiation upon lentiviral transduction of the wild type or dominant negative mutant forms of the BCL11A XL. Results are shown as mean ± SEM from three biological replicates. **e,** Western blots showing the specificity of BCL11A, γ-globin, GAPDH antibodies on day 12 of erythroid differentiation. Black and red arrows indicate different protein molecular weights in gel electrophoresis. Results are shown as mean ± SEM from three biological replicates. **f,** HbF flow cytometry on day 13 of HSPC erythroid differentiation. HbF: hemoglobin F (fetal form). Results are shown as mean ± SEM from three biological replicates. **g,** FACS analysis of CD49d, CD71 and CD235a erythroid markers on day 12 of HSPC erythroid differentiation. Results are shown as mean ± SEM from three biological replicates.

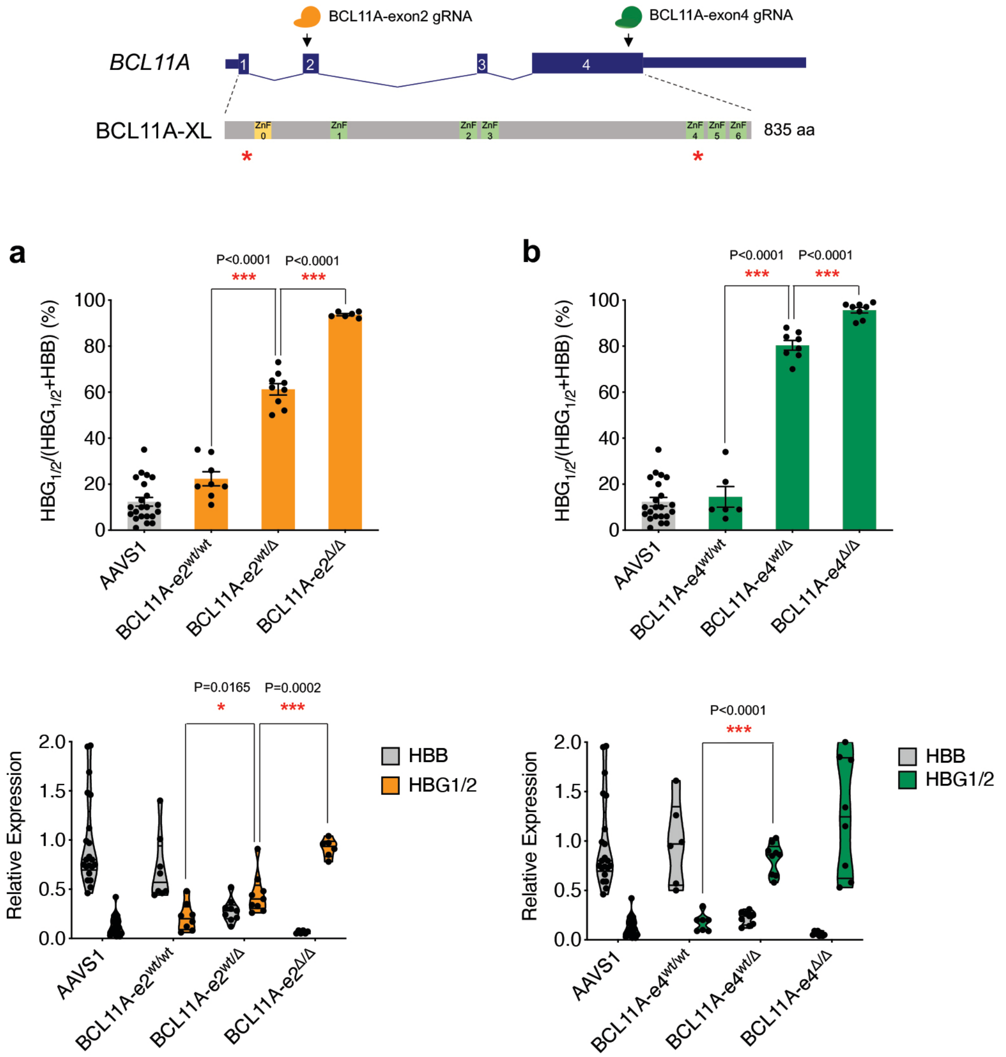

Supplementary Figure 9. Single cell-derived colony analysis through perturbation of BCL11A. a, Gene expression analysis for *HBG1/2* and *HBB* in HSPC-derived BFU-E upon genome editing of BCL11A exon 2, n=3 biologically independent experiments. Results are shown as mean ± SEM (P-values are labeled on the top of each comparison. *P<0.05, ***P<0.001 by a two-tailed Student t-test). **b,** Globin gene expression analysis in BFU-E upon genome editing of BCL11A exon 4, n=3 biologically independent experiments. Results are shown as mean ± SEM (P-values are labeled on the top of each comparison. ***P<0.001 by a two-tailed Student t-test).

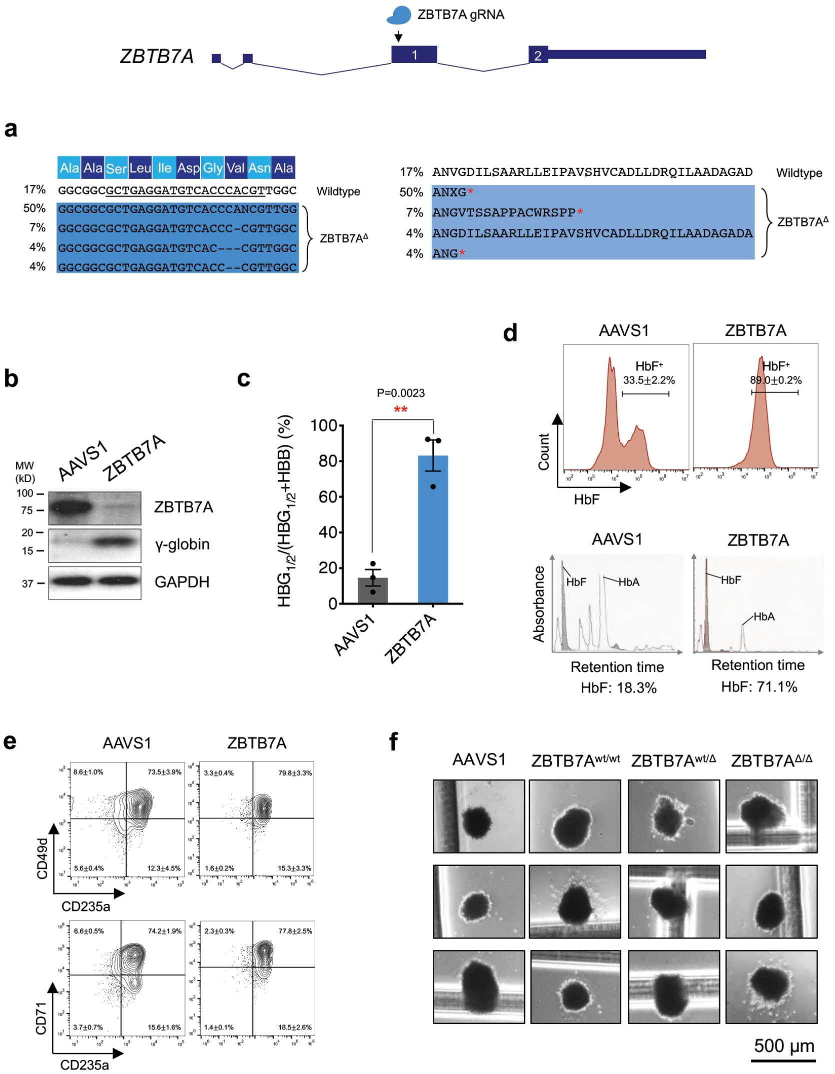

Supplementary Figure 10. Perturbation of ZBTB7A. a, Spectrum of genetic mutations and protein truncations in bulk HSPCs upon genome editing of ZBTB7A. ZBTB7A$^{\Delta}$: frameshift mutation in ZBTB7A. A red star indicates premature termination of protein elongation. **b,** Western blots showing the specificity of ZBTB7A, γ-globin, GAPDH antibodies in bulk HSPCs upon genome editing of AAVS1 and ZBTB7A. n=3 experiments were repeated independently with similar results. **c,** RT-qPCR analysis of γ-globin mRNA for HSPC erythroblasts on day 12 of erythroid differentiation upon genetic perturbations of AAVS1 and ZBTB7A. Results are shown as mean ± SEM from three biological replicates. (P-values are labeled on the top of each comparison. **P<0.01 by a two-tailed Student t-test). **d,** HbF flow cytometry on day 13 of HSPC erythroid differentiation and HPLC on day 17. HbF: hemoglobin F (fetal form); HbA0: hemoglobin A0 (adult form). HbF%: HbF as a fraction of HbA0 plus HbF. Results are shown as mean ± SEM from three biological replicates. **e,** FACS analysis of CD49d, CD71 and CD235a erythroid markers on day 11 of HSPC erythroid differentiation. Results are shown as mean ± SEM from three biological replicates. **f,** Morphology of BFU-E colonies for AAVS1, ZBTB7A$^{wt/wt}$, ZBTB7A$^{wt/\Delta}$, and ZBTB7A$^{\Delta/\Delta}$. n=3 experiments were repeated independently with similar results. Scale bar = 500 μm.

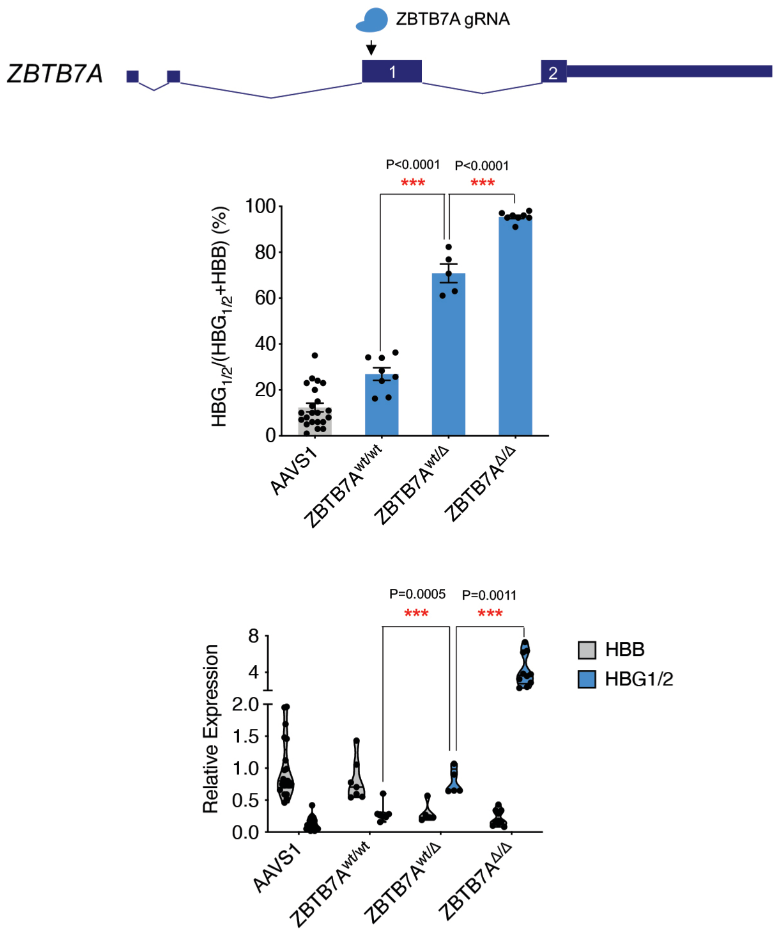

Supplementary Figure 11. Single cell-derived colony analysis through perturbation of ZBTB7A. Globin gene expression analysis in BFU-E upon genome editing of ZBTB7A, n=3 biologically independent experiments. Results are shown as mean ± SEM (P-values are labeled on the top of each comparison. ***P<0.001 by a two-tailed Student t-test).

ZBTB7A	BCL11A (e2&e4)	HBD-3.5kb	HBB-HBD	HBG1-13bp	HBG2-13bp
Δ/Δ	Δ/Δ	+/+	+/+	+/+	+/+
Δ/Δ	+/Δ	+/+	+/+	+/+	+/+
Δ/Δ	+/+	Δ/Δ	Δ/Δ	+/+	+/+
Δ/Δ	+/+	+/Δ	+/Δ	+/+	+/+
Δ/Δ	+/+	+/+	+/+	+/+	+/+
+/Δ	Δ/Δ	+/+	+/+	+/+	+/+
+/Δ	+/Δ	+/+	+/+	+/+	+/+
+/Δ	+/+	Δ/Δ	Δ/Δ	+/+	+/+
+/Δ	+/+	+/Δ	+/Δ	+/+	+/+
+/Δ	+/+	+/+	+/+	+/+	+/+
+/+	Δ/Δ	Δ/Δ	Δ/Δ	+/+	+/+
+/+	Δ/Δ	+/Δ	+/Δ	+/+	+/+
+/+	Δ/Δ	+/+	+/+	Δ/Δ	Δ/Δ
+/+	Δ/Δ	+/+	+/+	Δ/Δ	+/+
+/+	Δ/Δ	+/+	+/+	+/Δ	Δ/Δ
+/+	Δ/Δ	+/+	+/+	+/Δ	+/Δ
+/+	Δ/Δ	+/+	+/+	+/Δ	+/+
+/+	Δ/Δ	+/+	+/+	+/+	Δ/Δ
+/+	Δ/Δ	+/+	+/+	+/+	+/Δ
+/+	Δ/Δ	+/+	+/+	+/+	+/+
+/+	+/Δ	Δ/Δ	Δ/Δ	+/+	+/+
+/+	+/Δ	+/Δ	+/Δ	+/+	+/+
+/+	+/Δ	+/+	+/+	+/Δ	+/Δ
+/+	+/Δ	+/+	+/+	+/+	Δ/Δ
+/+	+/Δ	+/+	+/+	+/+	+/Δ
+/+	+/Δ	+/+	+/+	+/+	+/+
+/+	+/+	Δ/Δ	Δ/Δ	+/+	+/+
+/+	+/+	Δ/Δ	+/+	+/+	+/+
+/+	+/+	+/Δ	+/Δ	+/+	+/+
+/+	+/+	+/Δ	+/+	+/+	+/+
+/+	+/+	+/+	Δ/Δ	+/+	+/+
+/+	+/+	+/+	+/Δ	+/+	+/+
+/+	+/+	+/+	+/+	Δ/Δ	Δ/Δ
+/+	+/+	+/+	+/+	Δ/Δ	+/Δ
+/+	+/+	+/+	+/+	Δ/Δ	+/+
+/+	+/+	+/+	+/+	+/Δ	Δ/Δ
+/+	+/+	+/+	+/+	+/Δ	+/Δ
+/+	+/+	+/+	+/+	+/+	Δ/Δ
+/+	+/+	+/+	+/+	+/+	+/Δ
+/+	+/+	+/+	+/+	+/+	+/+

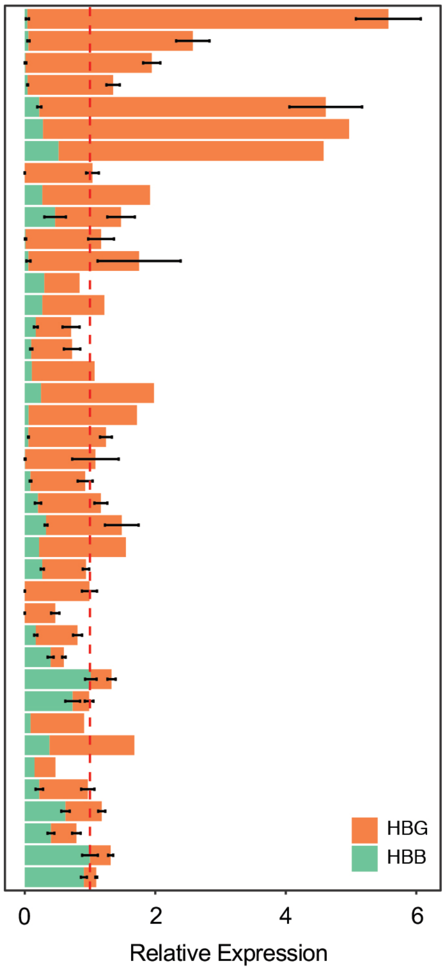

Supplementary Figure 12. Globin gene expression analysis across all genome-editing perturbation groups. At least n=3 biologically independent experiments were performed for each genome-editing perturbation group. Results are shown as mean ± SEM.

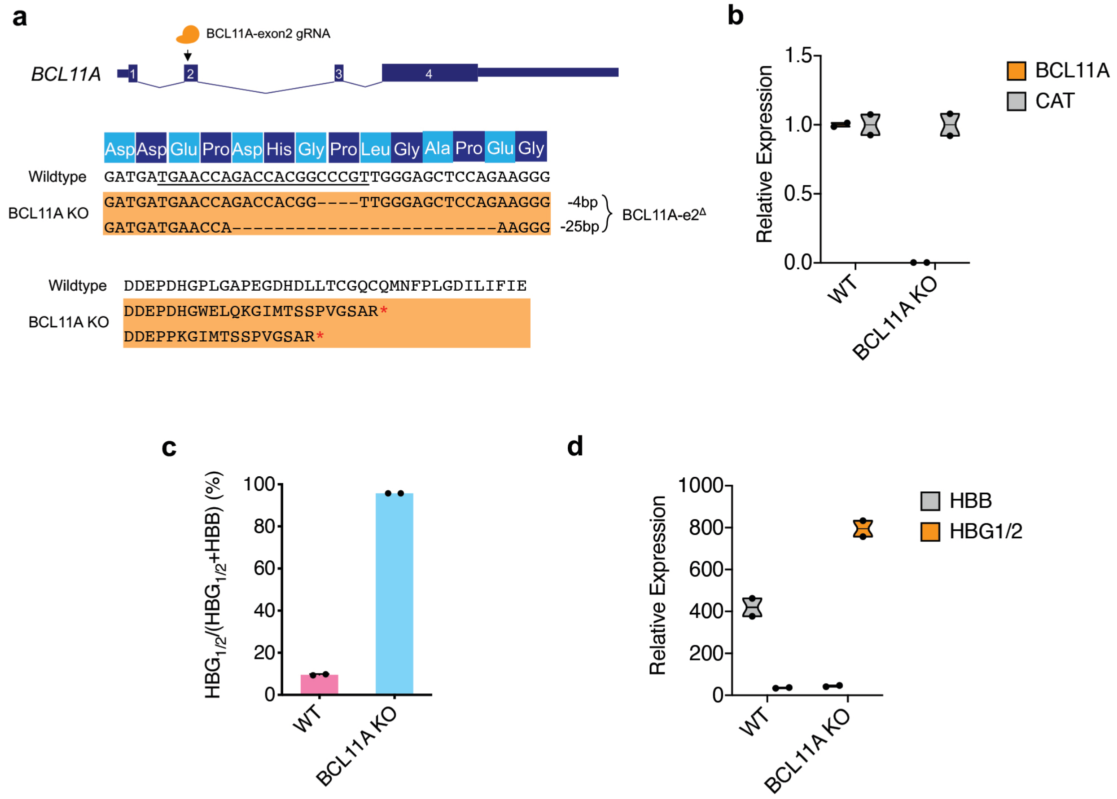

Supplementary Figure 13. HUDEP-2 BCL11A KO clone used in CAPTURE. a, Spectrum of frameshift mutations leading to protein truncations for HUDEP-2 BCL11A KO clone. **b-d,** RT-qPCR analysis of BCL11A and γ-globin mRNA for WT and BCL11A KO HUDEP-2 cells, n=2 biologically independent experiments. Results are shown as mean ± SEM (c) and violin plot (b, d). The gene encoding catalase (CAT) is used as a control for the expression shown in (b).

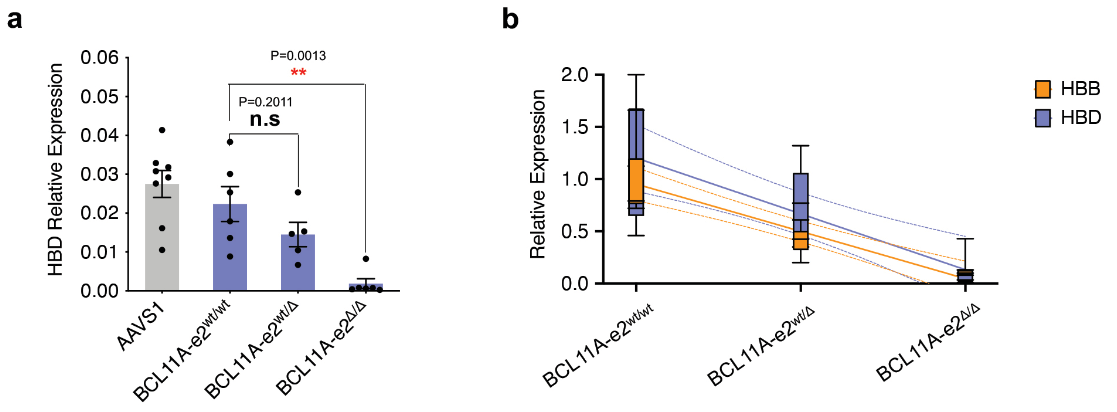

Supplementary Figure 14. Reduced *HBD/HBB* expression is associated with *BCL11A* editing mutations. a, RT-qPCR analysis of *HBD* mRNA for HSPC erythroblasts on day 12 of erythroid differentiation upon genetic perturbations of AAVS1 and BCL11A exon 2, n=3 biologically independent experiments. Results are shown as mean ± SEM (P-values are labeled on the top of each comparison. **P<0.01, n.s.: statistically non-significant by a two-tailed Student t-test). b, Linear fitting for *HBD/HBB* expression upon BCL11A$^{wt/wt}$, BCL11A-e2$^{wt/\Delta}$ and BCL11A-e2$^{\Delta/\Delta}$. Results are shown as box and whiskers plots including the min, first quartile, median, third quartile, and maximum.
