# Supplementary material for: A unified model of human hemoglobin switching through single-cell genome editing
Source: Nat Commun. 2021 Aug 17;12:4991. doi: 10.1038/s41467-021-25298-9 (PMC8371164; doi:10.1038/s41467-021-25298-9)
Supplement: Supplementary file 3 — Description of Additional Supplementary Files [file 41467_2021_25298_MOESM3_ESM.pdf]

Description of Additional Supplementary Files

File Name: Supplementary Data 1
Description: Fetal hemoglobin levels and cases of HPFH SNVs and the Δ13bp deletion within the proximal γ-globin (HBG1/2) promoters.

File Name: Supplementary Data 2
Description: SRA accession number of RNA-seq, ATAC-seq, ChIP-seq and CUT&RUN shown in Supplementary Figure 2.

File Name: Supplementary Data 3
Description: gRNA nucleotide sequence information.

File Name: Supplementary Data 4
Description: Primer sequences used for PCR screening and Sanger sequencing of gRNA editing efficiency.

File Name: Supplementary Data 5
Description: qPCR primer sequences used for detection of genome editing events.

File Name: Supplementary Data 6
Description: qPCR primer sequences used for mRNA expression.

File Name: Supplementary Data 7
Description: qPCR primer sequences used for 3C.

File Name: Supplementary Data 8
Description: Oligos for Site Directed Mutagenesis of BCL11A exon 4 mutations.

File Name: Supplementary Data 9
Description: Western blot antibodies

File Name: Supplementary Data 10
Description: Flow cytometry antibodies.
