# Supplementary material for: A unified model of human hemoglobin switching through single-cell genome editing
Source: Nat Commun. 2021 Aug 17;12:4991. doi: 10.1038/s41467-021-25298-9 (PMC8371164; doi:10.1038/s41467-021-25298-9)
Supplement: Supplementary file 14 — Reporting Summary [file 41467_2021_25298_MOESM14_ESM.pdf]

nature research

Corresponding author(s): Vijay G. Sankaran

Last updated by author(s): Jul 15, 2021

Reporting Summary

Nature Research wishes to improve the reproducibility of the work that we publish. This form provides structure for consistency and transparency in reporting. For further information on Nature Research policies, see our Editorial Policies and the Editorial Policy Checklist.

Statistics

For all statistical analyses, confirm that the following items are present in the figure legend, table legend, main text, or Methods section.

n/a	Confirmed	
☐	☒	The exact sample size (n) for each experimental group/condition, given as a discrete number and unit of measurement
☐	☒	A statement on whether measurements were taken from distinct samples or whether the same sample was measured repeatedly
☐	☒	The statistical test(s) used AND whether they are one- or two-sided<br>*Only common tests should be described solely by name; describe more complex techniques in the Methods section.*
☐	☒	A description of all covariates tested
☐	☒	A description of any assumptions or corrections, such as tests of normality and adjustment for multiple comparisons
☐	☒	A full description of the statistical parameters including central tendency (e.g. means) or other basic estimates (e.g. regression coefficient) AND variation (e.g. standard deviation) or associated estimates of uncertainty (e.g. confidence intervals)
☐	☒	For null hypothesis testing, the test statistic (e.g. F, t, r) with confidence intervals, effect sizes, degrees of freedom and P value noted<br>*Give P values as exact values whenever suitable.*
☒	☐	For Bayesian analysis, information on the choice of priors and Markov chain Monte Carlo settings
☒	☐	For hierarchical and complex designs, identification of the appropriate level for tests and full reporting of outcomes
☒	☐	Estimates of effect sizes (e.g. Cohen's d, Pearson's r), indicating how they were calculated

Our web collection on statistics for biologists contains articles on many of the points above.

Software and code

Policy information about availability of computer code

Data collection	Linear mixed modelling was performed using the R package lme4 (version 1.1-27). Data were visualized in R using the sjPlot (version 2.8.8) and ggplot2 (version 3.3.5) packages.
Data analysis	Data were analyzed using Microsoft Excel (version 16.16), GraphPad Prism 7, FlowJo (version 10.3), ImageJ (version 1.8.0), R (version 3.4.3).

For manuscripts utilizing custom algorithms or software that are central to the research but not yet described in published literature, software must be made available to editors and reviewers. We strongly encourage code deposition in a community repository (e.g. GitHub). See the Nature Research guidelines for submitting code & software for further information.

Data

Policy information about availability of data

All manuscripts must include a data availability statement. This statement should provide the following information, where applicable:

- Accession codes, unique identifiers, or web links for publicly available datasets
- A list of figures that have associated raw data
- A description of any restrictions on data availability

Source data are provided with this paper. The original data for globin gene expression analysis in BFU-E has been provided. Raw immunoblots for Supplementary Figure 7b, 8d, 10b are available online.

Field-specific reporting

Please select the one below that is the best fit for your research. If you are not sure, read the appropriate sections before making your selection.

☒ Life sciences ☐ Behavioural & social sciences ☐ Ecological, evolutionary & environmental sciences

For a reference copy of the document with all sections, see nature.com/documents/nr-reporting-summary-flat.pdf

Life sciences study design

All studies must disclose on these points even when the disclosure is negative.

Sample size	To integrate minimal donor-to-donor variability, at least 3 independent human donors are used for each genetic perturbation. 3 independent biological replicates were generally used for most experiments. To account for donor and other experimental sources of variation when applicable, as many BFU-E colonies as possible (usually 6~10) for each genetic mutation type were picked for downstream analysis.
Data exclusions	Data were not excluded from analysis.
Replication	To ensure the reproducibility of experimental findings, at least 3 replicates were performed. Experiments involving human erythroid progenitors were repeated with at least 3 independent donors to ensure consistency. To account for experimental sources of variation when applicable, as many BFU-E colonies as possible (usually 6~10) for each genetic mutation type were picked for downstream analysis. Data from all attempts at replication were included into analysis.
Randomization	At least 3 independent healthy HSPC donors were chosen randomly for each genetic editing type from the Fred Hutchinson Hematopoietic Cell Processing and Repository (Seattle, USA). BFU-E colonies were picked randomly for analysis among numerous colonies within each BFU-E pooling plate for each genetic mutation type.
Blinding	No blinding was performed in this study.

Reporting for specific materials, systems and methods

We require information from authors about some types of materials, experimental systems and methods used in many studies. Here, indicate whether each material, system or method listed is relevant to your study. If you are not sure if a list item applies to your research, read the appropriate section before selecting a response.

Materials & experimental systems

n/a	Involved in the study	
☐	☒	Antibodies
☐	☒	Eukaryotic cell lines
☒	☐	Palaeontology and archaeology
☒	☐	Animals and other organisms
☒	☐	Human research participants
☒	☐	Clinical data
☒	☐	Dual use research of concern

Methods

n/a	Involved in the study	
☒	☐	ChIP-seq
☐	☒	Flow cytometry
☒	☐	MRI-based neuroimaging

Antibodies

Antibodies used	Details (supplier name, catalog number, clone name) of all antibodies used are listed in Supplementary Tables 9 & 10. The dilution used for each antibody is mentioned in the methods section of the manuscript.
Validation	All antibodies in this study were used according to manufacturer's instructions. Species and applications have been provided here:<br>BCL11A Host species: Mouse, Tested applications: WB, Flow Cyt;<br>ZBTB7A Host species: Armenian hamster, Tested applications: WB;<br>Hemoglobin γ Host species: Mouse, Tested applications: WB, IP, IF, IHC(P) and FCM;<br>GAPDH Host species: Rabbit, Tested applications: WB, IP and IF.

Eukaryotic cell lines

Policy information about cell lines

Cell line source(s)	HUDEP-2 cell line was obtained from RIKEN BioResource Center, Tsukuba, Ibaraki, Japan.<br>Souce reference: Kurita R, Suda N, Sudo K, Miharada K, Hiroyama T, Miyoshi H, Tani K, Nakamura Y. Establishment of immortalized human erythroid progenitor cell lines able to produce enucleated red blood cells. PLoS One. 2013;8:e59890.<br>293T cell line was obtained from ATCC biorepository.

Authentication	Identity of cell lines were validated by STR analysis.
Mycoplasma contamination	Mycoplasma testing was routinely performed on all cells used in the study, and confirmed to test negative.
Commonly misidentified lines (See ICLAC register)	None of the cell lines used are listed in the ICLAC database.

Flow Cytometry

Plots

Confirm that:

- [x] The axis labels state the marker and fluorochrome used (e.g. CD4-FITC).
- [x] The axis scales are clearly visible. Include numbers along axes only for bottom left plot of group (a 'group' is an analysis of identical markers).
- [x] All plots are contour plots with outliers or pseudocolor plots.
- [x] A numerical value for number of cells or percentage (with statistics) is provided.

Methodology

Sample preparation	Cell cultures were harvested by centrifugation (500g, 5min), washed with phosphate-buffered saline (PBS, Invitrogen:14190-250) and stained with anti-human antibodies on ice for 20 min. All antibodies used are listed in Supplementary Table 3. Propidium iodide (PI) or 4,6-diamidino-2- phenylindole (DAPI) were used for live/dead cell discrimination.
Instrument	Flow cytometry analyses were carried out on Becton Dickinson (BD) LSRII, or Accuri C6 (BD) instruments.
Software	All data was analyzed using FlowJo software (version 10.3).
Cell population abundance	0.3-5 million post-sorted samples were analysed, depending on the experiment.
Gating strategy	Debris were initially removed with the FSC/SSC gating strategy. Live/dead discrimination was then carried out with DAPI/PI stains. Where applicable, these cells were then assessed for the expression of CD71 and CD235a surface markers, and gated based on the unstained control used in the experiment.

- [x] Tick this box to confirm that a figure exemplifying the gating strategy is provided in the Supplementary Information.
