# Supplementary material for: A unified model of human hemoglobin switching through single-cell genome editing
Source: Nat Commun. 2021 Aug 17;12:4991. doi: 10.1038/s41467-021-25298-9 (PMC8371164; doi:10.1038/s41467-021-25298-9)

# Source Data for Supplementary Figure 7b

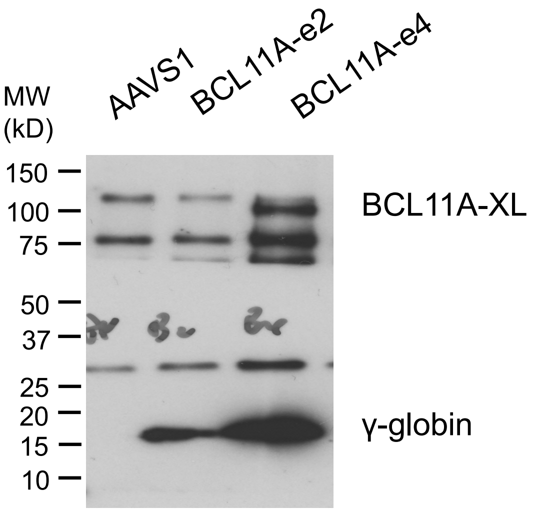


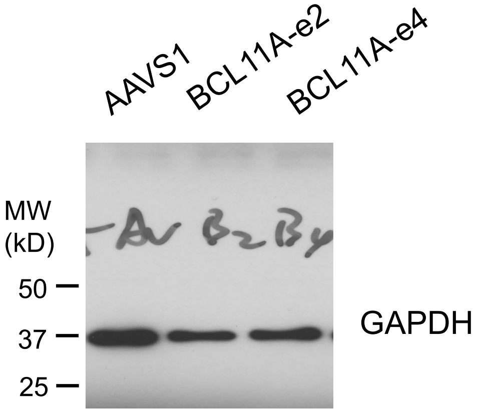

# Source Data for Supplementary Figure 8d

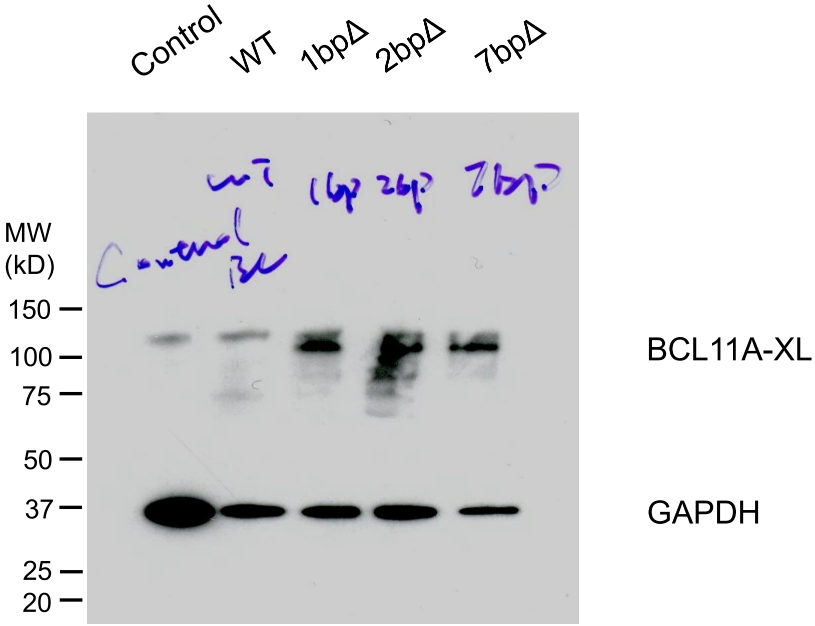


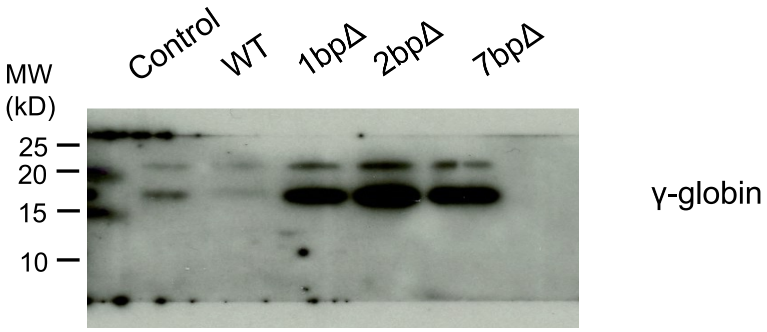

## Source Data for Supplementary Figure 10b

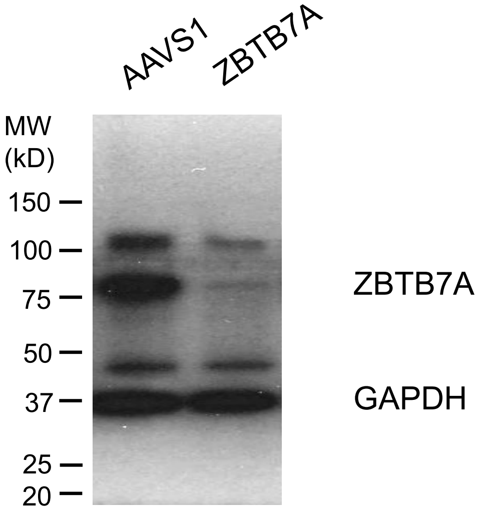


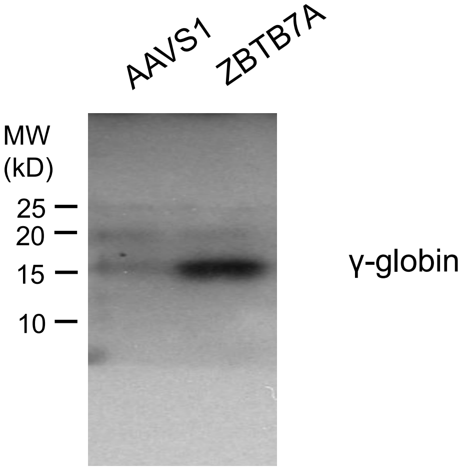

Supplement: Supplementary file 15 — Source Data [file 41467_2021_25298_MOESM15_ESM.zip › Souce Data/Source Data for raw immunoblots.pdf]
